# The protective effect of the spleen in sickle cell patients. A comparative study between patients with asplenia/hyposplenism and hypersplenism

**DOI:** 10.3389/fphys.2022.796837

**Published:** 2022-08-29

**Authors:** Sari Peretz, Leonid Livshits, Etheresia Pretorius, Asya Makhro, Anna Bogdanova, Max Gassmann, Ariel Koren, Carina Levin

**Affiliations:** ^1^ Pediatric Hematology Unit, Emek Medical Center, Afula, Israel; ^2^ The Ruth and Bruce Rappaport Faculty of Medicine, Technion–Israel Institute of Technology, Haifa, Israel; ^3^ Red Blood Cell Research Group, Vetsuisse Faculty, Institute of Veterinary Physiology, University of Zurich, Zürich, Switzerland; ^4^ Department of Physiological Sciences, Faculty of Science, Stellenbosch University, Stellenbosch, South Africa; ^5^ The Zurich Center for Integrative Human Physiology (ZIHP), Zürich, Switzerland

**Keywords:** hypersplenism, asplenia, fetal hemoglobin, reticulocytes, sickle cell disease, hyposplenism

## Abstract

Sickle cell disease (SCD) is caused by a point mutation in the beta-globin gene. SCD is characterized by chronic hemolytic anemia, vaso-occlusive events leading to tissue ischemia, and progressive organ failure. Chronic inflammatory state is part of the pathophysiology of SCD. Patients with SCD have extremely variable phenotypes, from mild disease to severe complications including early age death. The spleen is commonly injured in SCD. Early splenic dysfunction and progressive spleen atrophy are common. Splenomegaly and hypersplenism can also occur with the loss of the crucial splenic function. Acute, life-threatening spleen-related complications in SCD are well studied. The association of laboratory parameters with the spleen status including hyposplenism, asplenia, and splenomegaly/hypersplenism, and their implication in vaso-occlusive crisis and long-term complications in SCD remain to be determined. We evaluated the association between the spleen status with clinical and laboratory parameters in 31 SCD patients: Group a) Patients with asplenia/hyposplenism (N = 22) (including auto-splenectomy and splenectomized patients) vs. Group b) patients with splenomegaly and or hypersplenism (N = 9). Laboratory studies included: Complete Blood Count, reticulocyte count, iron metabolism parameters, C Reactive Protein (CRP), Hb variant distribution, and D-dimer. Metabolic and morphological red blood cell (RBC) studies included: density gradient (by Percoll), glucose consumption, lactate release, and K^+^ leakage, fetal RBC (F-Cells) and F-Reticulocytes, annexinV+, CD71^+^, oxidative stress measured by GSH presence in RBC and finally Howell Jolly Bodies count were all analyzed by Flow Cytometry. Scanning electron microscopy analysis of RBC was also performed. Patients with asplenia/hyposplenism showed significantly higher WBC, platelet, Hematocrit, hemoglobin S, CRP, D-dimer, Gamma Glutamyl Transferase (GGT), cholesterol, transferrin, annexin V+ RBCs, CD71^+^ RBCs, together with a markedly lower F Reticulocyte levels in comparison with splenomegaly/hypersplenism patients. In summary, important differences were also found between the groups in the studied RBCs parameters. Further studies are required to elucidate the effect of the spleen including hyper and hypo-splenia on laboratory parameters and in clinical manifestations, vascular pathology, and long-term complications of SCD. The benefits and risks of splenectomy compared to chronic transfusion need to be evaluated in clinical trials and the standard approach managing hypersplenism in SCD patients should be re-evaluated.

## Introduction

Sickle cell disease (SCD) is the most frequent worldwide hereditary hemoglobinopathy (Piel et al., 2013; Ware et al., 2017). The disorder is caused by the inheritance of abnormal beta-globin alleles carrying the sickle mutation on the HBB gene (HBB: c:20T>A- Glu6Val, βS). SCD results either by the homozygous state HbSS or by the combination of HbS with another abnormal HBB variants, mostly β^+^ or β^0^ thalassemia, both resulting in sickle red blood cells (RBC) (Bain 2008; Ware et al., 2017). Polymerization of deoxy-HbS generates abnormal dense and dehydrated RBC that play a central role in the acute and chronic manifestations of SCD. Intravascular sickling leads to impaired blood flow and vaso-occlusion with ischemic/reperfusion injury (Franceschi et al., 2011). Some organs, such as the spleen, are prone to be specifically vulnerable and damaged from HbS polymerization in SCD patients at early life (Platt 2000). Hyposplenism, asplenia, and progressive spleen atrophy (auto-splenectomy) are common. On the other hand, hypersplenism is characterized by an enlarged spleen (splenomegaly) which causes rapid and premature destruction of blood cells, resulting in thrombocytopenia, anemia, and or leukopenia, (Laws et al., 1979; Lv et al., 2016; Ladu et al., 2021). Splenomegaly and or hypersplenism can coexist with loss of function (Pearson 1969; O’Brien et al., 1976; Eichner 1979; Pearson et al., 1979; Serjeant et al., 1979; Zago et al., 1980; Brown et al., 1994).

In SCD, splenic dysfunction and hypersplenism are regularly observed during infancy; followed, in most cases, by spleen atrophy. In those cases, after repeated episodes of infarctions, the spleen is reduced to a fibrotic nodule (Diggs and Memphis 1935; Pearson et al., 1979). Another common and dangerous complication of SCD in young children is the sudden enlargement of the spleen defined as acute splenic sequestration crisis, associated with rapid decrease in hemoglobin levels of at least 2 g/dl, without signs of hemolysis (Lesher et al., 2009; Samuk et al., 2019).

Despite several postoperative complications, surgical splenectomy is recommended to treat the effects of hypersplenism in SCD subjects (Emond et al., 1984; Kyaw et al., 2006; Leone and Pizzigallo 2015; Iolascon et al., 2017; Luu et al., 2019; Tahir et al., 2020; Yacobovich et al., 2020).

One of the indicators for splenic dysfunction is the presence of Howell-Jolly bodies (HJB) inside RBCs. HJB are DNA inclusions that are usually formed at low frequency. Normally RBC containing HJB are removed from the peripheral blood by the spleen. In SCD patients, with functional asplenia, HJB are present in a higher amount than in healthy individuals. In addition to complete blood count (CBC), the presence of HJB is used as a splenic dysfunction marker (Harrod et al., 2007; El Hoss et al, 2019). Those results can provide a fast evaluation of spleen status.

Maintenance of normal spleen function is crucial in the prevention of morbidity and mortality in SCD patients. Since the spleen is involved in immune response and the clearance of senescent and aberrant blood cells, noninvasive, quantitative, and qualitative measurements of spleens’ function are required. In this study, we compared blood parameters and RBC properties of SCD patients and compared the results between SCD patients with asplenia, to patients with splenomegaly/hypersplenism. In addition, we implemented novel, non-routinely used methods for the evaluation of the functional status of the spleen in those patients.

## Materials and methods

### Patients

The study included 31 SCD patients treated at the Pediatric Hematology Unit, Emek Medical Center, (EMC), Afula, Israel. To get a better understanding of the spleen influence in SCD, the studied group was divided into two subgroups: patients with asplenia/hyposplenism [group (a)] which included patients with non-visualization of the spleen on ultrasound or no palpable spleen or patients after surgical total splenectomy, In this sub group we included young patients with splenic abnormal function and patients that probably underwent auto-splenectomy, both conditions well known in patients with SCD and patients that underwent surgical splenectomy. The second group, [group (b)], included patients with splenomegaly and/or hypersplenism. This group included patients with an enlarged spleen, palpable by physical examination and/or detected by ultrasound, with or without cytopenias, persistent for more than 6 months of follow-up.

The asplenia/hyposplenism group (group a) included 22 patients: 12 females; mean age 25.1 ± 12.9 years; 12 HbSS genotype and 9 HbS/β-genotype [1- β^+^, 8- β^0^ and another one probably β^0^ based on High-performance liquid chromatography (HPLC) results], one HbS/D-genotype and seven patients after surgical total splenectomy. The hypersplenism group (group b) included 9 patients: 6 females; mean age 16.8 ± 10.3 years (4 HbSS and 5 HbS/β-genotype (4- β^0^ and one- β^+^), patient characteristics are presented in (Table 1). RBC membrane permeability and metabolic properties studied in 14 healthy individuals and, Percoll density gradients studied in 21 healthy individuals were used as control values for comparison with the results of the study groups.

**TABLE 1:** Patients’ characteristics. Demographic and clinical features of 31 SCD patients enrolled in this study and divided into two groups: Asplenia/hyposplenism and Hypersplenism. Events of VOC and hospitalizations presented here are per year prior to the study.

	Asplenia/hyposplenism *n* = 22	Hypersplenism *n* = 9
	Average ± STD	Average ± STD
Age (years)	25.1 ± 12.9	16.8 ± 10.3
Gender female/male	12/10	6/3
Hb SS Genotype	12	4
Hb S/β Genotype	10	5
Splenectomyzed	7	—
Acute events VOC/study year^a^	10 (45%)	2 (22%)
Hospitalizations (admissions)^b^	14 (63%)	4 (44%)

a VOC: Asplenia/hyposplenism- 10 events (1 patient had 3 crises, 2 patients had 2 crises and another 2 had one crisis each); hypersplenism- 2 events (1 patient had 2 crisis).

b Hospitalizations: Asplenia/hyposplenism- 14 events (2 patients had 3 hospitalizations, 2 patients had 2 hospitalizations and 4 patients had one hospitalization each); hypersplenism- 4 events (2 patients had 2 hospitalizations).

All subjects were under stable doses of hydroxyurea treatment (500–2,000 mg/daily, 22.8 ± 9.6 mg/kg with no significant differences between the cohorts). Any of the patients were receiving routine blood transfusions or sporadic blood transfusions for at least 3 months before the study’s tests. Informed consent was obtained from all patients or their parents and the study received the approval of the local Helsinki Committee (Registry Nos. EMC-0071-17 and EMC-0120-17).

### Laboratory tests

Patients’ complete blood count (CBC) and blood biochemistry were analyzed on Advia2120 analyzer (*SIEMENS, Germany*) and AU5800 (*Beckman Coulter*, United States) and Clinical Chemistry Set-ups, respectively, at the EMC laboratory within 1 h after collection. High-performance liquid chromatography (HPLC) analysis of hemoglobin variant distribution was performed by Variant II analyzer (BIO-RAD, United States). The presented normal ranges are as accepted at the EMC, if not indicated differently.

### Separation on percoll density gradient levels

A whole blood sample was fractionated on a Percoll-based gradient as described (Makhro et al., 2013) with some modifications. Briefly, whole blood sample was layered over a Percoll solution (90% commercial Percoll (17-0891-01, GE Healthcare) and 10% of the concentrated plasma-like medium (1.4 M NaCl, 40 mM KCl, 7.5 mM MgSO4, 100 mM glucose, 0.15 mM ZnCl2, 2 mM Glycine, 2 mM Sodium Glutamate, 2 mM Alanine, 1 mM Arginine, 6 mM Glutamine, 1% BSA, 200 mM HEPES imidazole, pH 7.4) supplemented with 2 mM CaCl2, and then centrifuged at 18,514 g for 60 min (minimal acceleration/deceleration) at 30°C (Eppendorf Centrifuge 5810R, F-34-6-38 Rotor supplemented with specific adapter for 15 ml Falcon tubes). Then, the distribution of blood components was captured by the 16 Mp camera (installed in the Samsung Galaxy S6 Model SM-G920F mobile phone (Samsung Electronics Co.) and further analyzed by the free Windows version of the ImageJ software (downloaded from https://imagej.nih.gov/ij/download.html). The total visualized fraction was divided to equal ten stations and the intensity of each subfraction was measured.

### Glucose consumption, lactate release, and K+ leakage studies

Isolated RBC samples were incubated in the plasma-like medium (140 mM NaCl, 4 mM KCl, 0.75 mM MgSO4, 10 mM glucose, 0.015 mM ZnCl2, 0.2 mM Glycine, 0.2 mM Sodium Glutamate, 0.2 mM Alanine, 0.1 mM Arginine, 0.6 mM Glutamine, 0.1% BSA, 20 mM HEPES imidazole, pH 7.4) supplemented with 2 mM CaCl2 as described previously (Makhro et al., 2017) for 4 h at 37°C in gentle shake (14,000 rpm). The medium’s concentrations of potassium, glucose, and lactate were measured prior (time 0 h s) and after incubation (4 h s) by GEM^®^ Premier™ 4,000 blood gas analyzer. The 4 vs. 0 h s difference was normalized with total hemoglobin concentration measured simultaneously.

### Red blood cells flow cytometry studies

Morphological characteristics of RBC and reticulocytes, phosphatidylserine (PS) exposure on outer membrane leaflet representing early apoptotic RBCs was measured by annexin V+ RBCs, intracellular Ca^2+^ and oxidative stress measured by reduced glutathione (GSH) levels in RBC were investigated by FC as described elsewhere (Kuypers et al., 1996; Kämpf et al., 2019). Briefly, 2 µl of whole blood suspended in a 1 ml plasma-like medium was loaded with 1.5 µl of either:

- Intracellular Ca^2+^ dye, Fluo-4 AM [1 mM stock, (Thermo Fisher Scientific].
- GSH binding dye, monobromobimane (MBBR) (100 mM stock, Thermo Fisher Scientific).
- Anti-CD71-FITC (TfR) antibody (IQP-152F, IQ Products) for identification of reticulocyte fraction.
- eBioscience™ Annexin V Apoptosis Detection Kit eFluor 450 dye (Thermo Fisher Scientific) for PS externalization test.

The stained cells were incubated for 45 min in dark before FC measurements.

RBC sizes and morphology were examined in unstained samples.

In addition, the presence of DNA inclusions inside the RBCs, the Howell-Jolly bodies—HJB) (El Hoss et al., 2018) and fetal RBC (F-Cells), F Reticulocytes (representing early changes in HbF cells), were also examined using FC. HbF and F-Cells are used to monitor SCD patients’ response to Hydroxyurea treatment and to evaluate their chances to suffer from SCD-related complications (Lebman et al., 1982; Kono et al., 2009; Sato et al., 2010).

In brief, 20 μl of whole blood in an EDTA tube fixed in 1 ml of 25% glutaraldehyde solution (*Sigma*, United States) for 10 min. Immediately after fixation, 100 μl of the suspension was permeabilized in a new tube by resuspension in 400 μl of 0.1% TRITON X-100 (*Sigma*, United States) for 4 min. Following permeabilization, 20 μl of the suspension was taken in a new tube with 5 μl of 5 μM Hoechst 33342 (*Sigma*, United States), 5 μl 7-AAD (7-amino-actinomycin D) (*Invitrogen*, United States), 5 μl anti CD71 (transferrin receptor) APC conjugate (*Invitrogen*, United States), 5 μl intercellular anti-human fetal Hb (MHFH01) FITC conjugate (*Life technologies*, United States) and 70 μl PBS. After vortex and dark incubation for 15 min, 400 μl of PBS was added and gentle vortex before FC measurement.

The fluorescence intensity was measured on RBC gate using Navios EX flow cytometer (*Beckman Coulter*, United States). Double measurements (>50,000 total cells/measurement) were performed and averaged for each condition. All data were analyzed using Kaluza Analysis Software (*Beckman Coulter*, United States https://www.beckman.co.il/flow-cytometry/software/kaluza).

### Scanning electron microscopy imaging of blood components

10 μl of whole blood was pipetted into a 10 mm round glass microscope cover slip and dried for 2 min at room temperature. The glass microscope cover slips were placed in a 24-well culture flask for further washing; PBS was added and removed after 15 min. Next, 4% formaldehyde was added to the sample for fixation for a minimum of 30 min., After three washes with PBS one drop of osmium tetra-oxide (OT) (*Sigma*, United States) was added directly onto the cover slip and then covered with dH2O for 15 min. Right after, the samples were washed three times with PBS for 3 min each wash. Next, dehydration was performed by a series of Ethanol-dH2O mixtures (30%, 50%, 70%, 90%, and finally 100%) for 3 min. The last wash with 100% ethanol was repeated twice. At this stage, the well plate was sealed with parafilm, and the samples were stored for 24–48 h. Next, the samples were covered with 99.9% hexamethyldisilazane (HMDS) (*Sigma*, United States) for 30 min. Then, the open plate was left overnight to evaporate HMDS. The prepared samples were coated with carbon and viewed with a Zeiss MERLIN field emission-SEM with the InLens detector using 1 kV (Carl Zeiss Microscopy, Munich, Germany).

### Statistics

Data for the entire study was analyzed using GraphPad 5 software. Differences between SCD patients with Asplenia/hyposplenism vs. Hypersplenism were tested using the Mann-Whitney test. For all analyses, a two-tailed test with *p* < 0.05 was accepted as statistically significant.

## Results

### Patient laboratory characteristics

Comparison of the CBC and biochemistry parameters revealed significant differences between hypersplenic and asplenic/hyposplenic SCD subjects (Table 2).

**TABLE 2:** CBC and biochemistry parameters in asplenic/hyposplenic compared to hypersplenic SCD subjects. Normal ranges are presented in accordance with the criteria accepted by the EMC Lab divisions. The data is presented as average ± Standard Deviation (SD); Significance was calculated using the two-tailed Mann-Whitney as *p* < 0.05. WBC, White blood count, ANC, Absolute Neutrophil count, ALC, Absolute lymphocyte count, AMC, Absolute monocyte count, AEC, Absolute eosinophil count, ABC, Absolute basophil count, Hb, Hemoglobin, HCT, Hematocrit, Hypo, Hypochromic Red Cells, PLT, Platelet, RET: Reticulocyte, HbF, Fetal hemoglobin, HbS, S hemoglobin, LDH, Lactic dehydrogenase, GGT, Gamma Glutamyl Transferase, CRP, C reactive protein. RBC, Red blood cells.

Parameter (Units)	Normal range	Asplenia/Hyposplenism *n* = 22^a^	Hypersplenism *n* = 9^a^	*p*-value
WBC (K/μl)	4.5–11.5	10.06 ± 3.568	4.674 ± 2.093	0.0002
ANC (K/μl)	1.5–6	5.526 ± 2.416	2.486 ± 1.007	0.0005
ALC (K/μl)	1.5–6	3.160 ± 1.193	1.719 ± 0.987	0.0031
AMC (K/μl)	0.1–0.8	0.705 ± 0.536	0.237 ± 0.213	0.0005
AEC (K/μl)	0–0.8	0.287 ± 0.168	0.090 ± 0.038	0.0009
ABC (K/μl)	0–0.2	0.078 ± 0.044	0.017 ± 0.017	0.0001
Hb (g/dl)	M:14–17; F: 12–15	9.718 ± 1.279	8.006 ± 0.949	0.0025
HCT (%)	M: 40–54; F: 37–47	29.83 ± 3.968	25.02 ± 2.508	0.0036
Hypo (%)	0–2.5	6.609 ± 5.471	12.70 ± 6.968	0.0313
PLT K/µl	150–450	501.2 ± 194.3	156.2 ± 54.48	<0.0001
RET absolute (K/µl)	20–100	208.0 ± 86.37 (19)	181.1 ± 88.87 (9)	ns
HbF (%)	0.5–1.5	17.67 ± 7.185	22.26 ± 8.156	ns
HbS (%)	0	71.42 ± 11.01	59.87 ± 12.54	0.0124
D-Dimer (ng/ml)	0–500	1451 ± 990.4 (14)	439.2 ± 83.17 (5)	0.0014
Albumin (g/dl)	3.5–5.2	4.328 ± 0.310 (21)	4.131 ± 0.168 (9)	0.0374
LDH (U/L)	230–480	783.6 ± 353.2	937.0 ± 325.8	ns
Cholesterol (mg/dl)	<200	120.2 ± 23.16 (21)	83.28 ± 12.54 (9)	0.0004
GGT: (U/L)	M:0–55; F: 0–38	58.60 ± 73.04 (16)	9.944 ± 2.698 (5)	0.0028
Serum Potassium (mM/dl)	3.5–5.1	4.318 ± 0.286	3.868 ± 0.230	0.0006
Serum Calcium (mM/dl)	8.5–10.5	9.468 ± 0.3737	9.148 ± 0.1905	0.0066
Ferritin (ng/ml)	M:22–322; F: 10–291	579.1 ± 644.1	960.9 ± 1065	ns
Serum iron (µg/dl)	M:60–160; F: 40–145	103.8 ± 64.14	68.53 ± 29.12	ns
Transferrin (mg/dl)	200–360	240.9 ± 44.47 (17)	183.8 ± 23.92 (4)	0.0224
CRP (mg/dl)	0.00–0.50	1.445 ± 1.986 (20)	0.371 ± 0.341 (9)	0.0360

a Number of patients studied when not all the patients in this group were analyzed.

We found a significantly lower hematocrit and hemoglobin levels (approximately 2 g/dl reduction) in the hypersplenism vs. asplenia/hyposplenism group (*p* < 0.05) that was accompanied by an almost two-fold increase in the percentage of hypochromic RBC in the hypersplenism group (*p* = 0.03).

Lower white blood cell (WBC) and platelet counts were found in the hypersplenism group compared to the asplenia/hyposplenism group (*p* < 0.001), similarly to all the subsets of leukocytes (Table 2). The mean percent of HbF was higher in the hypersplenism group (an increase of 5%) but this difference was not significant. The mean percent of HbS was significantly lower in hypersplenism compared with the asplenia/hyposplenism group (*p* = 0.01).

The D-Dimer was significantly lower in the hypersplenism group but within the normal range, while in the asplenia/hyposplenism was increased (*p* < 0.05).

Serum Lactic dehydrogenase (LDH) levels were elevated in both groups, representing the chronic hemolytic state, with no differences between the groups (Table 2). Serum albumin, cholesterol, calcium, and potassium levels were within normal values in both groups but were significantly lower in the hypersplenism than in the asplenia/hyposplenism group (*p* = 0.05).

The CRP levels, representing the inflammation state, were within the normal range in the hypersplenism group, while in the asplenia/hyposplenism group were abnormally increased (*p* < 0.05).

No significant differences between the groups were found in ferritin and serum iron, however, the transferrin level was lower in the hypersplenism group (*p* < 0.05).

### Metabolic and morphological characteristics of red blood cells

**FIGURE 1:**
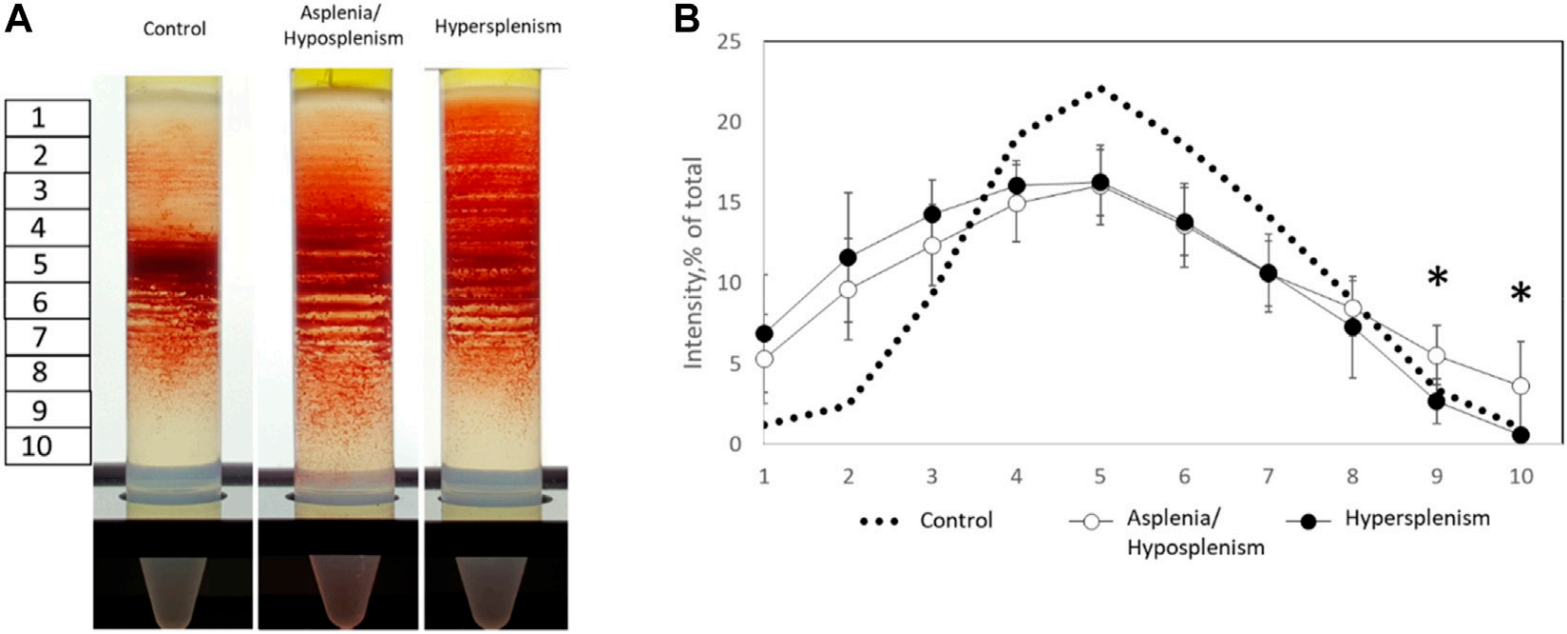
Percoll density gradients of control and SCD subjects for dense and hydrated RBC fractions. **(A)** Representative Percoll gradients for control, asplenic/hyposplenic, and hypersplenic subjects are demonstrated. **(B)** The RBC content in each station was estimated as a percentage from a sum of all sub-fraction intensity values. The correspondent examination of RBCs obtained from control subjects (*n* = 21; 10M/11F; 46.8 ± 14.2 years) was made. The data is presented in average ± SD; the significance is < 0.05.

To specify the RBCs-related differences between the groups, blood samples were fractionated on Percoll density gradient as described. We found a significant reduction of dense/senescent RBC fractions (lower stations 9 and 10) in the hypersplenism compared with the asplenia/hyposplenism group. This demonstrates that the hypersplenism group contains more hydrated and less dense/dehydrated and sickled RBCs when compared to the asplenia/hyposplenism group (Figure 1).

**FIGURE 2:**
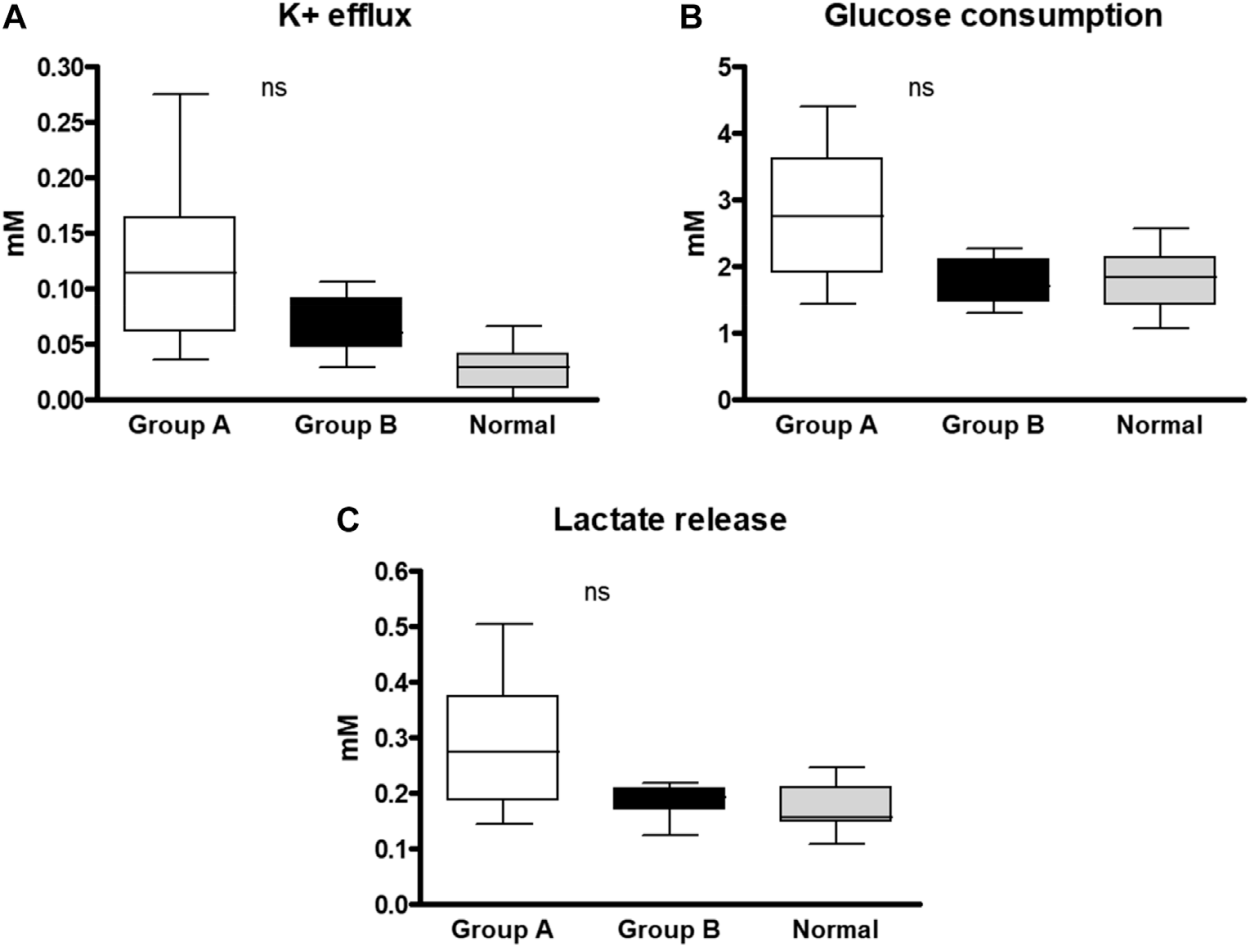
RBC membrane permeability and metabolic properties of SCD subjects and healthy control. Samples of each group were incubated for 4 h in a plasma-like medium and examined for: **(A)** K+ efflux, **(B)** Glucose consumption, and **(C)** Lactate release. The 4 vs. 0 h difference was normalized with total hemoglobin concentration. The correspondent examination of RBCs obtained from control subjects (*n* = 14; 8M/6F; 34.1 ± 16.1 years) was made. The data is presented in average ±SD; the significance is < 0.05. Group (A) patients with asplenia/hyposplenism, Group (B) patients with hypersplenism.

Our next target was to study RBC membrane permeability and metabolic properties (Figure 2). We found no significant difference in the RBCs membrane permeability between the two groups measured by K^+^ leak and lactate released from RBCs. Also, the RBCs glucose consumption. was similar in the two groups, however, the values in the hypersplenism group were lower than in the asplenia/hyposplenism group and approximates to normal values of healthy controls.

### Red blood cells flow cytometry characteristics and percentage HbF by high-performance liquid chromatography

**FIGURE 3:**
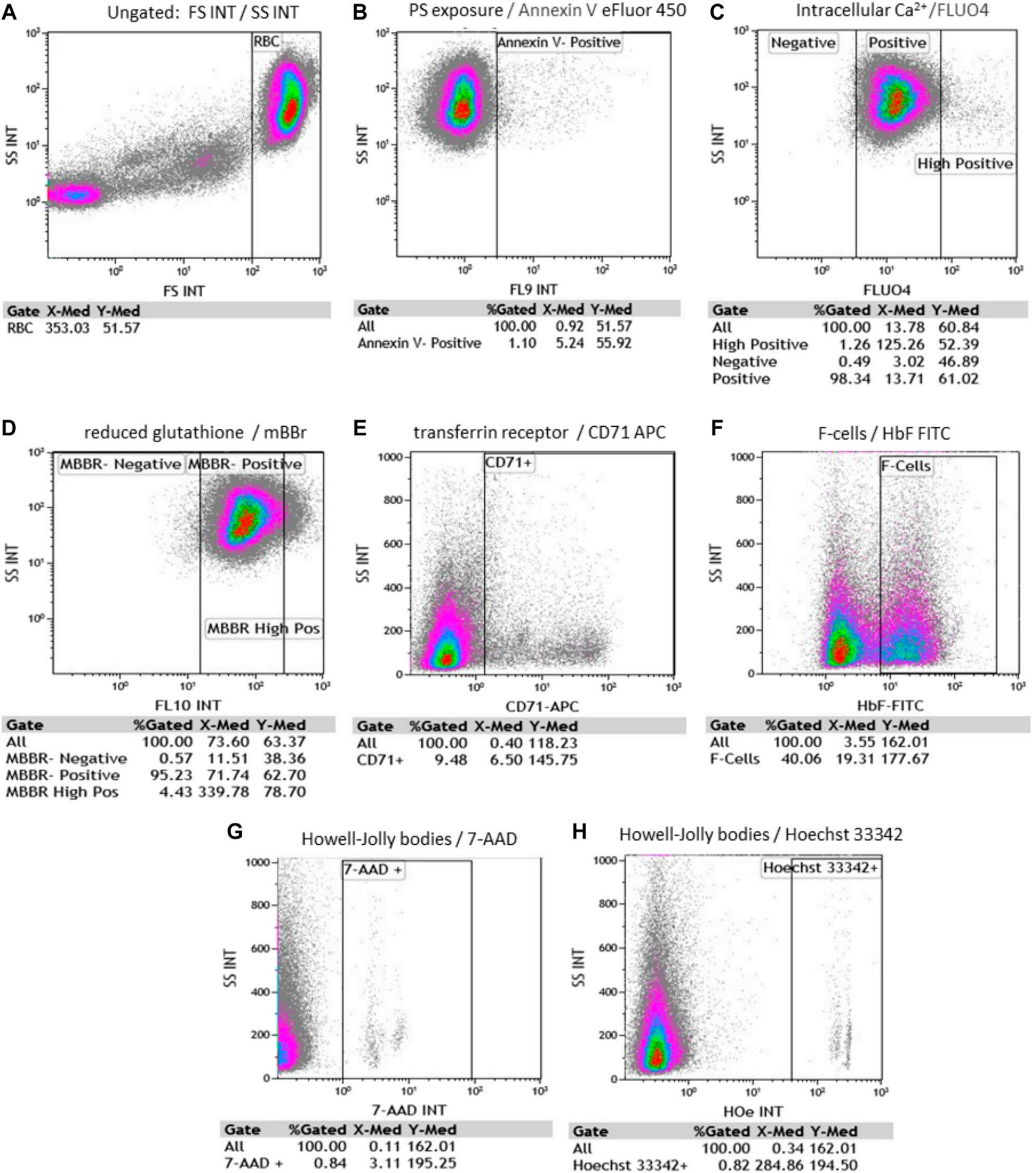
Representative flow cytometry scatter plots and gating strategy for RBC. Each evaluates % gated cells and median fluorescence intensity (MFI). **(A)** RBC side and forward scattering of unstained cells (SS and FS, accordingly); **(B)** Annexin V-positive RBC; **(C)** fluo-4 positive and fluo-4 high-positive RBC; **(D)** MBBR-positive and MBBR high-positive RBC; **(E)** CD71 positive RBC; **(F)** F- RBC (F-cells) positive fraction **(G)** 7-AAD (7-amino-actinomycin D) positive RBC and **(H)** Hoechst 33342 positive RBC. [**(B–H)** plots were all gated on RBC].

Using FC, we evaluated RBC and reticulocytes morphological characteristics, phosphatidylserine (PS) exposure on outer membrane leaflet, intracellular Ca^2+^, oxidative stress measured by reduced glutathione (GSH), reticulocytes identification, F- cells, and HJB levels. Representative scatter plots and gating strategy for RBC were shown for all fluorophores (Figure 3).

Side scatter (SSC) and forward scatter (FSC) median fluorescence intensity (MFI) of unstained RBC were 55.39 ± 5.6 and 399.6 ± 39.64; respectively in the hypersplenism group. and 64.29 ± 6.24 and 407.3 ± 45.89; respectively in the asplenia/hyposplenism group.

**FIGURE 4:**
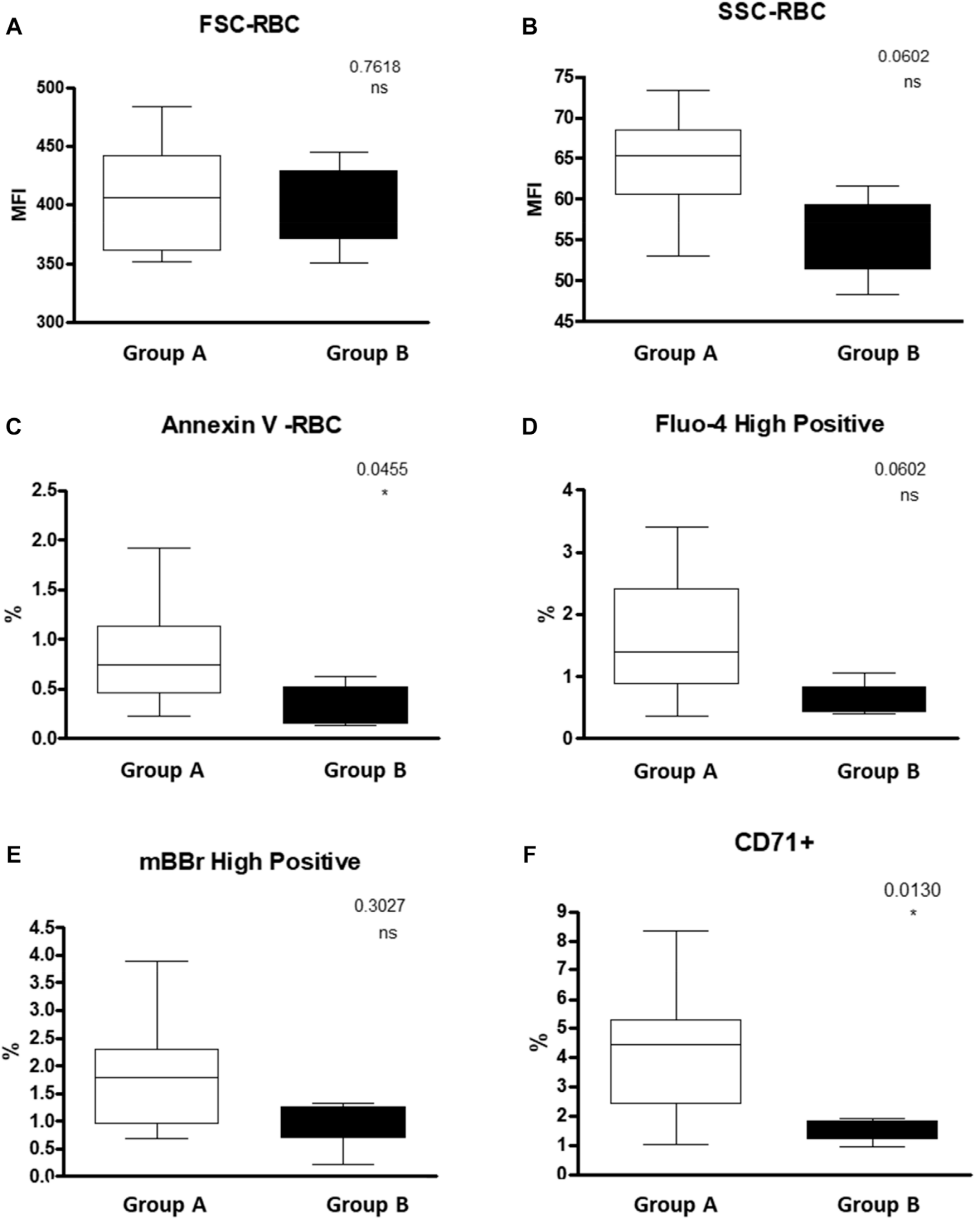
RBC properties of SCD patients Asplenic/hyposplenic compared to Hypersplenic using Flow cytometry: **(A)** + **(B)** FSC and SSC of RBC; **(C)** Annexin V positive RBC; **(D)** fluo-4 high-positive RBC; **(E)** MBBR high-positive RBC; **(F)** CD71 positive RBC. Group (A) patients with asplenia/hyposplenism, Group (B) patients with hypersplenism.

We found a lower percentage of Annexin V+ RBCs, in the hypersplenism group compared to the results in the asplenia/hyposplenism group (mean 0.34 ± 0.2% vs. 0.82 ± 0.5% respectively, *p* = 0.05). We also found lower, but not statistically significant, RBC intracellular Ca^2+^ levels as measured by fluo-4-high-positive RBCs, in the hypersplenism group compared to asplenia/hyposplenism **(**mean % 0.63 ± 0.3 vs. 1.63 ± 0.95; respectively *p =* ns. and MFI 96.56 ± 10.48 vs. 87.73 ± 44.88; respectively *p =* ns). Additionally, we observed decreased trend of MBBR-high-positive RBC in the hypersplenism group vs. the asplenia/hyposplenism group (mean % 0.97 ± 0.49 vs. 1.75 ± 0.93; respectively *p* = ns and MFI 356.66 ± 95.49 vs. 495.37 ± 107.81; respectively *p =* ns). Also, the total MBBR positive population MFI was lower in the hypersplenism group vs. the asplenia/hyposplenism group (mean MFI 68.23 ± 22.79 vs. 103.61 ± 28.04; respectively *p* = 0.04) (Figure 4).

We found that the percent of immature RBCs CD71^+^, reticulocytes expressing the transferrin receptor on the cell surface was lower in the hypersplenism vs. asplenia/hyposplenism group (mean % 1.5 ± 0.4 vs. 4.2 ± 2.1; respectively*, p* = 0.013). These results correlate with a decreased trend in total reticulocyte count in the CBC in the hypersplenism group (Figure 4).

**FIGURE 5:**
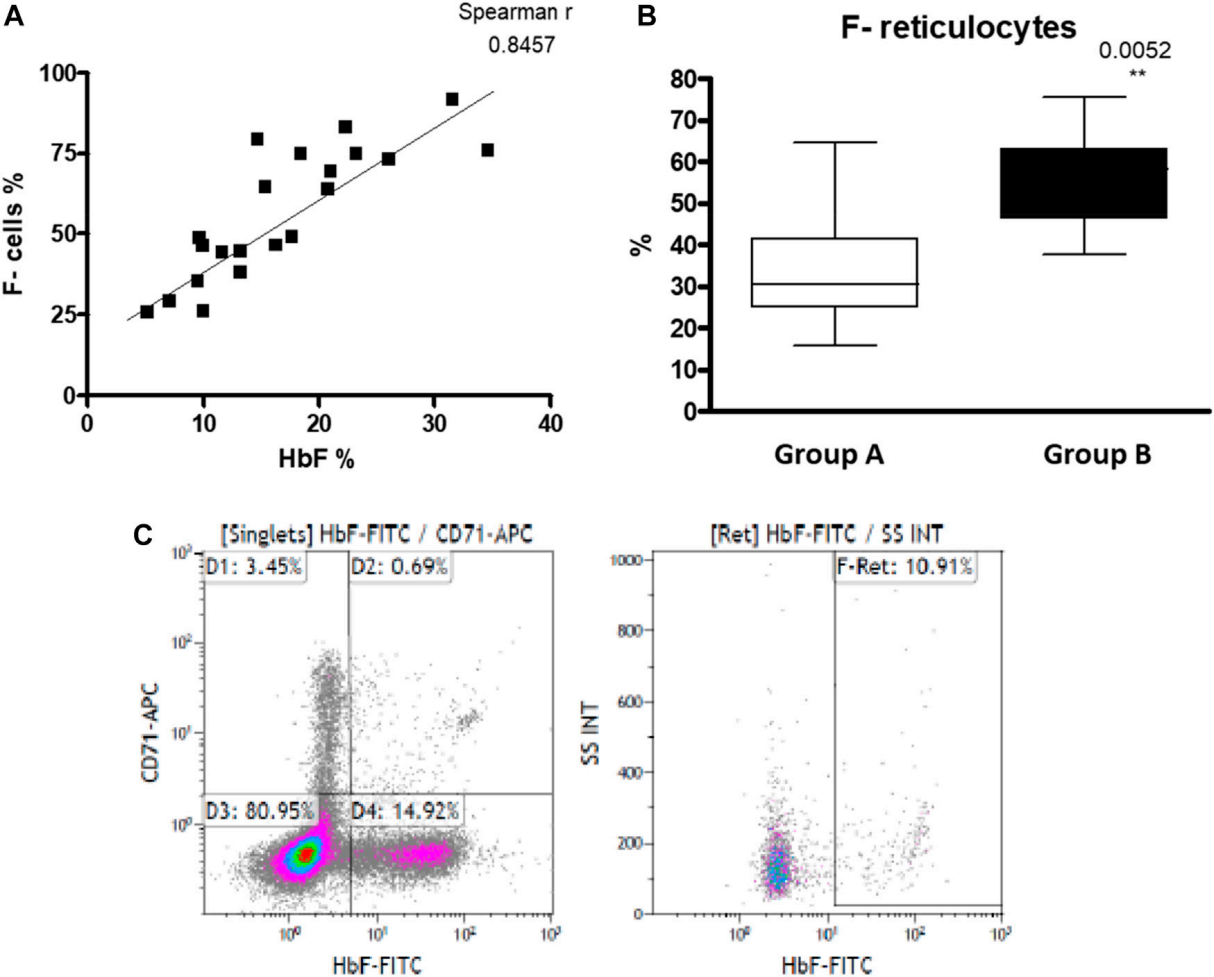
HbF in RBC of SCD patients: **(A)** HbF measured by HPLC and F- cells measured by FC positively correlates (*n* = 21). **(B)** F- reticulocytes count in asplenic/hyposplenic (*n* = 14) vs. hypersplenic (*n* = 7) SCD subjects. **(C)** representative FC gating strategy for F- Reticulocytes. Group (A) patients with asplenia/hyposplenism, Group (B) patients with hypersplenism.

We found a higher, but not statistically significant, level of HbF measured by HPLC in the hypersplenism group compared to asplenia/hyposplenism (mean HbF % 22.26 ± 8.15 vs. 17.67 ± 7.18; respectively *p =* ns.) (Table 2), and percent of F-Cells measured by FC (Mean % 64.01 ± 19 vs. 52.77 ± 20; respectively *p =* ns). We show a positive correlation between these two parameters (Spearman, *r* = 0.8457) (Figure 5). Since reticulocytes circulate in the peripheral blood for 7 days, the calculation of F- reticulocyte can serve as a unique and important value for patient’s follow-up and treatment response. We found significantly higher F-reticulocyte counts in the hypersplenism compared to the asplenia/hyposplenism group (mean 33.2 ± 14.1 vs. 55.1 ± 12.6%; respectively *p* = 0.011) (Figure 5).

**FIGURE 6:**
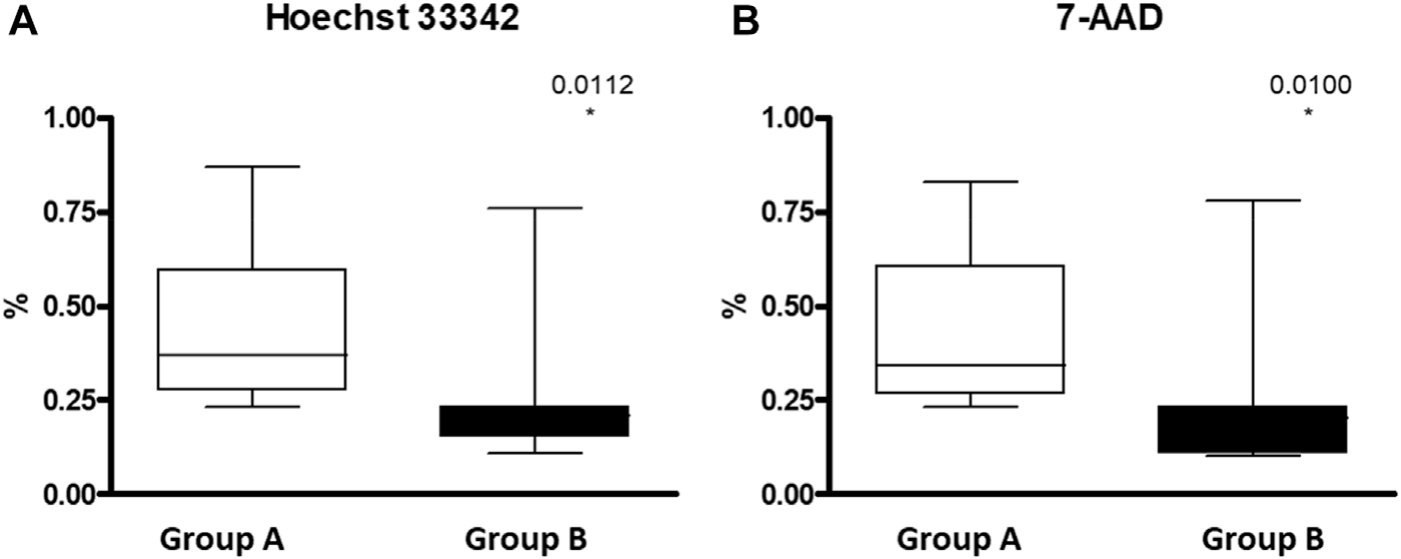
HJB inside RBC measured by FC in asplenic/hyposplenis vs. hypersplenic SCD subjects: **(A)** Hoechst 33342 for A and T nucleotides and **(B)** 7-AAD (7-amino-actinomycin D) for C and G nucleotides. Group (A) patients with asplenia/hyposplenism, (*n* = 13). Group (B) patients with hypersplenism, (*n* = 7).

Splenic dysfunction examined by the presence of HJB in the RBCs using FC revealed, as expected, a significantly lower count of HJB in the hypersplenism group compared to asplenia/hyposplenism patients in both nucleotides’ dyes; Hoechst 33342 and 7-AAD (0.27 ± 0.2 and 0.26 ± 0.2 vs. 0.43 ± 0.2 and 0.43 ± 0.2 respectively *p* = 0.01) (Figure 6).

**FIGURE 7:**
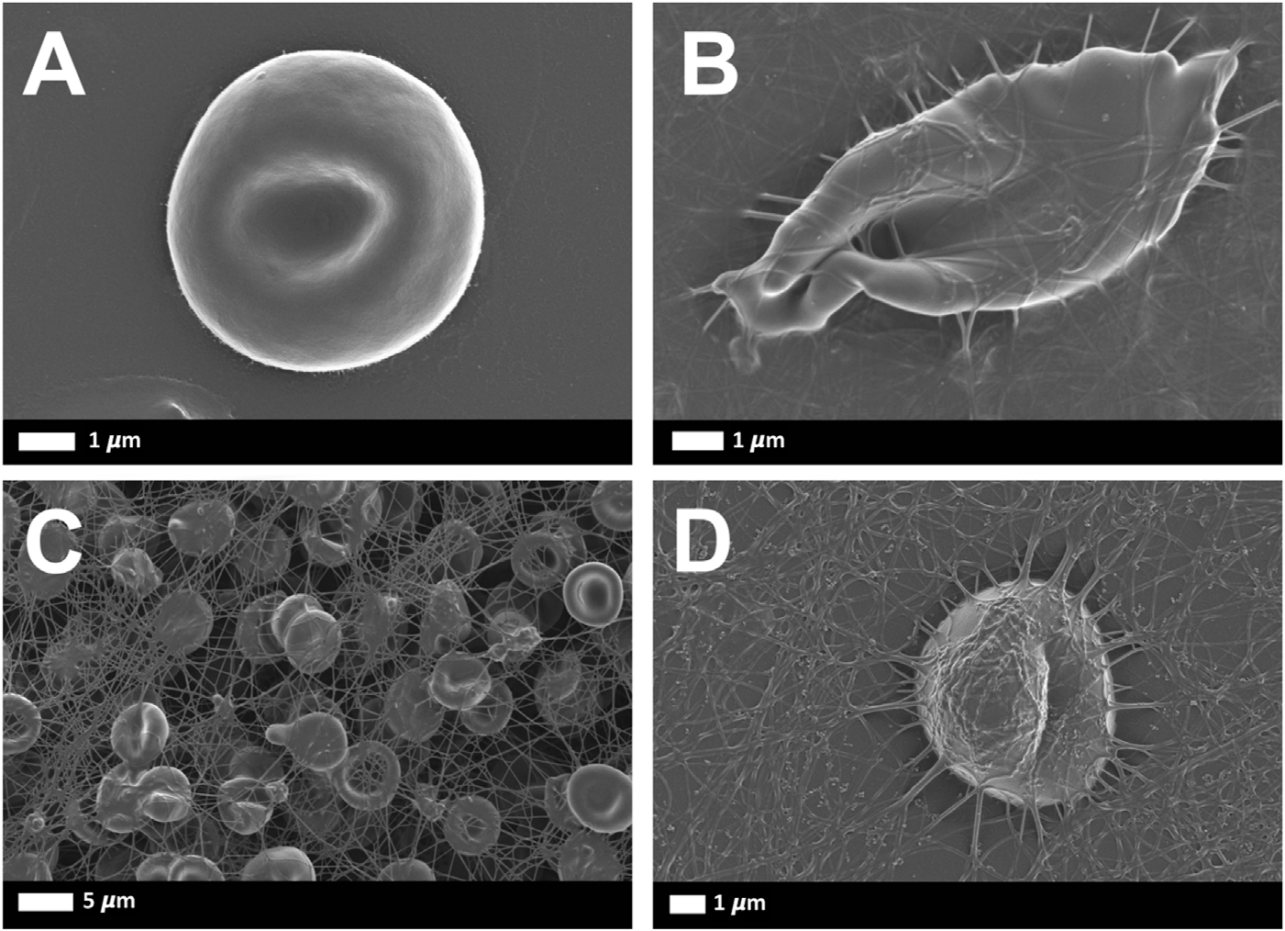
Representative scanning electron microscopy images of RBCs from healthy and SCD subjects: **(A)** Representative RBC from a healthy individual **(B)** Sickle RBC, **(C)** RBCs with fibrin net **(D)** single RBC with fibrin net that spontaneously formed.

Representative SEM images of RBCs from healthy and SCD subjects were shown including sickle shape RBC and RBCs with fibrin clot net in a patient with asplenia (Figure 7).

## Discussion

The spleen has several functions including clearance of damaged blood cells and antibodies production (Lewis et al., 2019). On the one hand, SCD patients without a spleen suffer from the presence of damaged RBC which can increase the risk of vaso-occlusive crises and overwhelming severe bacterial infections (el Hoss and Brousse, 2019). On the other hand, the negative consequences of hypersplenism are associated with the increased clearance of blood cells and blood-borne antigens, leading to cytopenia and some degree of immunodeficiency caused by the leukopenia (Brousse et al., 2014). Excessive destruction of RBCs in SCD children with prolonged hypersplenism may lead to subsequent growth impairment and bone marrow hyperplasia. Also, transfusion efficiency is usually reduced in SCD patients with hypersplenism (Laws et al., 1979; Lv et al., 2016; Ladu et al., 2021). One of the targets of our study was to use simple tests to monitor spleen status in SCD patients. For example, the use of DNA dyes and the easy handling FC method can serve as a good alternative for HJB count, instead of a labore microscope examination.

### Laboratory tests

The findings of the low Hb, HCT total WBC, WBC subtypes, and platelets in the hypersplenism group can be explained by spleen clearance of blood cells. On the other hand, the relative thrombocytosis in the asplenia/hyposplenism group is related to the lack of platelet clearance by the absent or nonfunctional spleen. Our results related to the blood cells counts confirm most of the previously reported data (al-Salem et al., 1996; Fasola and Adekanmi 2019; el Hoss and Brousse, 2019).

Lower percent of HbS in hypersplenism group compared with the asplenia/hyposplenism group, is probably related to the more effective clearance of sickled RBC by the spleen (Brousse et al., 2014)., It is well known that the HbS content and the percentage of HbF are the main modulators of clinical severity in SCD, and even small changes in sickle RBCs hemoglobin composition may reduce or delay polymerization with great significance on clinical manifestations (Hebert et al., 2020). The slightly higher mean percent of HbF and the F-Cells in the hypersplenism group suggest less Hb polymerization.

Chronic inflammatory state that is part of the pathophysiology of the SCD (Adekile 2013), we observed increased acute-phase reactant proteins in patients with non-functional spleen, the asplenia/hyposplenism group. Among those findings we can mention the higher D Dimer the lower transferrin that are common in anemia of chronic diseases (Costa et al., 2008), and the higher and abnormally elevated CRP and ferritin levels all observed in the asplenia/hyposplenism group.

Also, the high abnormal D-Dimer that we found in the asplenia/hyposplenism group probably indicated a prothrombotic state and not only a marker of inflammation. It is known that in a healthy individual, the main clotting plasma protein, fibrinogen, is in a soluble state. In those subjects’ fibrin network formation can be induced if thrombin is added to whole blood (Palta et al., 2014). Fibrin deposits that form without the addition of thrombin are only noted during pathological clotting (Lipinski et al., 2012). However, in the presence of circulating inflammatory molecules, in inflammatory conditions, the plasma proteins may be induced to form spontaneous fibrin networks, without the presence of thrombin. Since SCD is a chronic inflammatory state, fibrin production can be spontaneously seen (Figures 7C,D). Hypercoagulation and clotting pathologies are well-known accompaniments to inflammatory conditions (Pretorius et al., 2017; Olumuyiwa-Akeredolu et al., 2019; Randeria et al., 2019).

In the current study, we observed normal electrolyte values in our SCD patients, however, the serum potassium and calcium were lower in the hypersplenism group compared to the asplenia/hyposplenism, group probably reflecting reduced intravascular hemolysis. The increase in serum potassium has been described in sickle cell patients, with higher levels during an acute crisis (Pandey et al., 2012; Mansoor et al., 2021). In addition to an increased release caused by the chronic hemolysis, serum K^+^ abnormalities may be related to the status of hydration, the degree of acidosis during acute events, abnormalities in renal function, and alterations in RBCs ion transporting systems. Some of these RBCs systems such as the Gardos channel, K-Cl cotransporter, and the Na/K pump are directly involved in the maintenance of RBC hydration; their increased activity contributes to RBC dehydration in SCD subjects (Vitoux et al., 1989; Gibson et al., 1998; Izumo et al., 1987; Luthra and Sears 1982; Hannemann et al., 2015).

### Separation on percoll density gradient levels

Percoll density gradient was used as an indication of RBCs dehydration-rehydration state (Hänggi et al., 2014). The reduction of dense/senescent RBC fractions observed in patients with hypersplenism compared with the asplenia/hyposplenism group demonstrates that the hypersplenism group contains more hydrated and less sickled RBCs; which can be considered a beneficial effect related to increased clearance of abnormal RBCs by the spleen.

### Red blood cells flow cytometry studies: Oxidative stress

Nicotinamide adenine dinucleotide (NAD) is a ubiquitous oxidation-reduction (redox) cofactor in RBCs. The synthesis of NAD and its reduced form, NADH, play major roles in maintaining redox balance (Zerez et al., 1988). Sickle RBCs have a lower redox ratio [(NADH):(NAD+ + NADH)] than normal RBCs. Increased reduced glutathione (GSH) availability can decrease oxidative stress status and potentially reduce sickled RBC (Niihara et al., 2018). MBBR interaction with GSH forms a highly fluorescent derivative detected by FC. Here we showed decreased MBBR-high-positive RBC both percentage and MFI in the hypersplenism group compared with the asplenia/hyposplenism group. These results were contrary to what we expected and to the literature. One of our study limitations is the low sample size in both groups. We believe that increasing the sample size can reveal more genuinely the MBBR results and then facilitate explaining those facts. In addition, since reticulocytes contain increased amounts of reduced glutathione than mature RBC (Roth et al., 1979) and we found a 3-fold increase of reticulocytes count in the asplenia/hyposplenism group compared to hypersplenism, we hypothesis that the clearance of reticulocyte by the spleen of hypersplenic patients may have led to decreased MBBR-high-positive RBC which also included reticulocytes.

### Red blood cells flow cytometry studies: Morphology

Regarding the results of the flow cytometry analysis, as previously done (Warang et al., 2011; More et al., 2020), we used SSC and FSC values to assess the RBCs size.

Here we observed an interesting tendency for the RBC hypochromia and a decrease in RBC side scatter value, in patients with hypersplenism compared with the asplenia/hyposplenism group. This suggests on the one hand a reduced number of sickled and hyperchromic RBCs. And on the other hand, less senescent RBCs, normally removed by the spleen, appear to be selectively cleared by the spleen in patients with hypersplenism (Girasole et al., 2012).

This is in line with the significantly lower number of early apoptotic RBCs (annexin V+) in this patient group. Additionally, we show that these morphological changes appear in view of the decrease in the dense RBCs at lower fractions of Percoll density gradient separation as well as with a decrease in the number of RBCs high-positive intracellular Ca^2+^ concentrations. These findings confirm the previously reported data of higher intracellular Ca^2+^ content in dense RBCs than in light RBC fractions (Romero et al. 1997). Furthermore, such an increase of RBCs with elevated Ca^2+^ may be directly connected with the correspondent increase in PS-exposing erythrocytes as shown by Wesseling (Wesseling et al., 2016).

Normally, PS is confined to the cytoplasmic RBC membrane leaflet, PS external expression represents a signal for clearance and early apoptosis of RBCs, known to be increased in SC RBCs.(Hannemann et al., 2018; Thiagarajan et al., 2021; Anke). In accordance with the known evidence that PS externalization is realized by the scramblase activated by an increase of intracellular Ca^2+^ content (Wesseling et al., 2016), we demonstrate a lower percent of RBCs with abnormal elevated intracellular Ca^2+^ together with a significant reduction in annexin V + RBCs in hypersplenic subjects.

The fluctuation in intracellular Ca^2+^ and the increased externalization of PS in RBCs may also contribute to hypercoagulability and the pathological clotting that was noted in the SEM analysis (Figure 7) (Hood et al., 2018; Swieringa et al., 2018; Toorn et al., 2019).

### Red blood cells flow cytometry studies: F-cells

Another parameter used to monitor the severity and drug treatment response in SCD patients is the HbF level analyzed by HPLC, or the F-Cells measured by FC. Hydroxyurea (HU) treatment for SCD patients leads to an increase in HbF and the number of F-Cells RBC concomitant with the improvement of clinical symptoms (Hebert et al., 2020). Hydroxyurea is a safe and effective treatment for SCD, however a proportion of SCA patients are not responding to HU treatment (Pule et al., 2015), in any case, it is important to evaluate patient’s response to this treatment, titrate the dosage and prevent unnecessary overdose. It is known that the clinical response to HU treatment can be evident a few days after treatment initiation and on the opposite sudden stop of HU can provoke an acute pain crisis long before the HbF level drops (Pule et al., 2015).

Measurement of F Reticulocytes using FC, after a few days of HU treatment, will provide a fast understanding of each patient’s response to HU treatment instead of the routinely used HbF measurement analyzed by HPLC, which shows HbF changes usually after 3–4 months (Steinberg et al., 2014). In our study, we found that F Reticulocytes count was significantly higher in hypersplenic subjects compared to asplenia/hyposplenism. We found that the percent of immature RBCs CD71^+^, reticulocytes expressing the transferrin receptor on the cell surface was lower in the hypersplenism vs. asplenia/hyposplenism group. These results correlate with a decreased trend in total reticulocyte count in the CBC suggesting an accelerated maturation of reticulocytes in hypersplenic subjects and less hemolysis (Lebman et al., 1982; Kono et al., 2009). The impact of the number of circulating reticulocytes as well as its influence on sickling and oxidative stress mechanism in SCD patients need further research.

In patients with functional spleen and hypersplenism the enhanced clearance of sickled RBC (especially due to its pathological morphological and rheological properties), together with the specific slow and open microcirculation in the spleen provides the best platform to favor deoxygenation, promoting RBC sickling and subsequent, accelerating RBC destruction (Brousse et al., 2014). Thus, despite the improvement in RBC rheology and metabolism, these features may be counteracted with aggravation of the anemia and anemia-related complications.

One of the study limitations is the relatively small number of patients studied and more than that this fact did not allow comparison inside each subgroup. In addition, the results of our study should be compared with other clinical parameters like age and clinical severity. The patients with hypersplenism are younger than the patients with asplenia/hyposplenism but they are in the second decade of age, beyond the age when usually asplenia develops in SCD patients. We also suggest, that in the future, a study that will use the power of electron microscopy at single cell level, to make a robust comparative RBCs morphological evaluation of asplenia/hyposplenism vs hypersplenism.

In conclusion, in addition to the known laboratory manifestations of hypersplenism such as severe pancytopenia, our results show some significant “beneficial” laboratory findings including lower HbS, CRP, and D-dimmer values and lower morphological and metabolic pathological properties of the RBCs. The results of this study may provide other tools to define more precisely the spleen function in SCD patients. Given our results, it is extremely important to make any efforts to preserve the spleen in patients with SCD and preserve the immunological and hematological functions including the eradication of abnormal and senescent blood cells. New treatments should be focused on the prevention of functional asplenia and hypersplenism and avoid performing surgical splenectomy.

The benefits and risks of total splenectomy compared to chronic transfusion need to be evaluated in clinical trials and the standard approach to managing hypersplenism in SCD patients should be re-evaluated.

## Data Availability

The original contributions presented in the study are included in the article/Supplementary Material, further inquiries can be directed to the corresponding author.
